# Genome-wide identification, characterization, evolution and expression analysis of the *DIR* gene family in potato (*Solanum tuberosum*)

**DOI:** 10.3389/fgene.2023.1224015

**Published:** 2023-08-23

**Authors:** Wenqi Jia, Yuting Xiong, Man Li, Shengli Zhang, Zhongcai Han, Kuihua Li

**Affiliations:** ^1^ Agricultural College, Yanbian University, Yanji, Jilin, China; ^2^ Jilin Academy of Vegetable and Flower Science, Changchun, Jilin, China

**Keywords:** abiotic stress, bioinformatics, DIR, gene family, potato

## Abstract

The *dirigent* (*DIR*) gene is a key player in environmental stress response and has been identified in many multidimensional tube plant species. However, there are few studies on the *StDIR* gene in potato. In this study, we used genome-wide identification to identify 31 *StDIR* genes in potato. Among the 12 potato chromosomes, the *StDIR* gene was distributed on 11 chromosomes, among which the third chromosome did not have a family member, while the tenth chromosome had the most members with 11 members. 22 of the 31 *StDIRs* had a classical *DIR* gene structure, with one exon and no intron. The conserved DIR domain accounts for most of the proteins in the 27 *StDIRs*. The structure of the *StDIR* gene was analyzed and ten different motifs were detected. The *StDIR* gene was divided into three groups according to its phylogenetic relationship, and 22 duplicate genes were identified. In addition, four kinds of cis-acting elements were detected in all 31 *StDIR* promoter regions, most of which were associated with biotic and abiotic stress. The findings demonstrated that the StDIR gene exhibited specific responses to cold stress, salt stress, ABA, and drought stress. This study provides new candidate genes for improving potato’s resistance to stress.

## 1 Introduction

*Solanum tuberosum*, commonly known as potato, represents a crucial non-cereal food crop cultivated across approximately 19.3 million hectares globally, yielding an annual production of nearly 400 million metric tons, ranking only behind rice, wheat, and maize (Halterman et al., 2016; Liu et al., 2023). In recent years, people’s demand for potato is increasing day by day, and the demand for planting areas is also gradually increasing, but the resulting planting problems are also increasing significantly. These consequent problems caused a large reduction in potato production, resulting in great economic losses. To cope with severe biotic and abiotic stress, plants also spontaneously evolved a series of defense measures to quickly respond to the complex environment, reduce the damage and strive for more survival resources (Atkinson and Urwin, 2012; Zulfiqar and Ashraf, 2022). Plants respond to complex environments by activating many different genes, including the DIR protein (Arasan et al., 2013; Lopez-Zaplana et al., 2022; Meher et al., 2022).

The inception of *dirigent* (*DIR*) genes dates back to 1985, when they were initially characterized in pea (*Pisum sativum*). But the function of DIR proteins were first identified in *Forsythia intermedia* in 1997, and then was later reported in Arabidopsis (*Arabidopsis thaliana*) (Ralph et al., 2006; Cheng et al., 2018; Zhang et al., 2022; Luo et al., 2022). *DIR* belongs to a multi-gene family. DIR protein is relatively conserved, among which the conserved domain accounts for a large part of DIR protein, and usually does not contain introns in the *DIR* gene (Corbin et al., 2018). *DIR* genes are found in almost all vascular plants, including ferns, gymnosperms, and angiosperms (Arasan et al., 2013; Song and Peng, 2019; Ma et al., 2021). Furthermore, DIR has been demonstrated to play a guiding role in lignin synthesis (Cheng et al., 2018; Dong et al., 2021; Liu et al., 2021). Research has illustrated the congruence between the localization sites of DIR proteins and the locations of lignin deposition and biosynthesis (Davin and Lewis, 2000; Burlat et al., 2001). Thus, DIR proteins play a crucial role in directing the correct formation of lignin. Lignin is mainly deposited in terminally differentiated cells, which is an important part of the cell wall. Lignin is essential for resistance to adverse external environments, serving as the first physical barrier to safeguard plant growth and development. The involvement of DIRs in the biosynthesis of lignin units and lignin has attracted the scientific community to discover the functions of these proteins in various stress tolerance mechanisms.

*DIR exhibits responsiveness to a wide array of biotic and abiotic stresses*. At the same time, there have been many studies on *DIR*, most of them related to disease resistance and tolerance. *DIR* genes have been found in a variety of plants, and all of them play important roles. The results showed that overexpression of *GhDIR1* in cotton would lead to the increase of lignin content, which could improve the resistance of cotton to *Verticillium dahliae* (Shi et al., 2012). The loss of *CaDIR7* function reduced root activity after salt stress and inhibited the induction of stress-related genes in *CaDIR7* silenced plants. *CaDIR7* gene silencing in pepper significantly reduced the tolerance of pepper to pathogens and salt stress (Khan et al., 2018). There is evidence of the overexpression of *GmDIR22* in soybean can increase total lignan accumulation and enhance plant resistance to *Phytophthora sojae* (Li et al., 2017). The high expression of *DIR* in flax leads to pinoresinol accumulation after the pathogen invasion of plants (Corbin et al., 2018). In terms of resistance to insect and pathogen stresses induced by weevils or mechanical damage, six *DIR* genes from Sitka spruce (*Picea sitchensis*) bark can be expressed rapidly and strongly (up to 500-fold) (Ralph et al., 2007). Hormonal pathways hold significance within plant regulatory networks, and investigations have demonstrated that DIR likewise exhibits responsiveness to hormones. An earlier report showed that a *dirigent* gene (*BhDIR1*) from *Boea hygrometrica* was involved in response to plant dehydration status, applied phytohormones, signaling molecules and temperature stresses (Wu et al., 2009).

Currently, research on the potato DIR gene family is limited. Furthermore, investigations into DIR have insufficiently acknowledged the contribution of potato DIR genes in responding to both biotic and abiotic stresses. The study of the *StDIR* gene in potato can provide important information for the molecular breeding of potato. In this experiment, we analyzed the gene structure, chromosomal location, phylogenetic relationship family evolutionary tree and transcriptomic-based expression data of the potato *StDIR* gene family and constructed the expression map of this gene in different tissues under different biotic and abiotic stresses. This study conducted a detailed and comprehensive analysis of the *DIR* gene family, which could provide a reference for the research on the role of the *DIR* gene family in the process of exploring potato’s response to biotic and abiotic stresses.

## 2 Materials and methods

### 2.1 Plant materials and treatment

Germinated seed tubers (10 g) of potato variety Youjin were planted in a greenhouse of Yanbian University with day/night time is 16/8 h, and a day/night temperature of 23/17°C. The 50-days-old potato plants were treated with cold, salt, PEG, and ABA stress for 0, 24, 48, 72, and 96 h. The methods of treatment are followed: Cold: The culture temperature was 4°C, Salt: Place potato seedlings in a 200 mM NaCl solution, Drought: Place potato seedlings in a 10% polyethylene glycol (PEG, polyethylene glycol) solution and ABA (Abscisic Acid): The 0.1 mM ABA solution was applied evenly to the surface of the potato seedlings until the solution was about to drip from the edge of the leaves. The top leaves of each potato plant were taken and stored in a −80°C refrigerator for later use, and each treatment groups contain three biological replicates.

### 2.2 StDIR gene identification and chromosomal distribution analysis

First of all, the total proteins sequence file, the genome annotation file (GFF3 format) and the genome sequences file of potato (*Solanum tuberosum*) were downloaded from the Ensembl plants database (http://plants.ensembl.org/index.html). The amino acid sequence of 25 Arabidopsis DIR proteins was downloaded from TAIR (The Arabidopsis Information Resource, https://www.arabidopsis.org/) database and was used as blast queries against the total proteins file of potato. Then, the hidden Markov model (HMM) file of conserved DIR-like protein domain PF03018 was downloaded from the Pfam database (https://www.ebi.ac.uk/Tools/hmmer/search/phmmer). All the proteins sequence of the above two methods results were submitted to Pfam and CDD (Conserved Domains Database, https://www.ncbi.nlm.nih.gov/cdd) to confirm they contain the conserved domain of DIR protein. The gene ID, genomic location, and amino acid number were obtained from the genome annotation file. ProtParam software (https://web.expasy.org/protparam/) was used to analyze the molecular weight (MolWt) of proteins and the theoretical isoelectric point (pI). In the potato genome annotation file, the length information of potato chromosomes and the position information of all potato *DIR* genes on chromosomes were obtained. At the same time, TBtools was used to draw the chromosome localization map of potato *DIR* genes, which was used to represent the position information and distance relationship of all potato *DIR* gene family members on chromosomes (Chen et al., 2020).

### 2.3 StDIR genes structure, conserved domains and conserved motifs analysis

All StDIR protein sequences of the potato were obtained from the total protein sequence file of the potato and used as input files. Conserved motif analysis was performed using MEME v4.12 software. The parameters were set as follows: the number of conserved motifs was 10, the amino acid length of the smallest motif was 6, the amino acid length of the largest motif was 100, and the number of searches was 10,000. Gene structure analysis was performed using TBtools to identify the coding sequence (CDS) and untranslated region (UTR) of the *StDIR* gene. The conserved domain information of StDIR proteins was obtained from the CDD database Finally, TBtools was used to visualize the conserved motifs, conserved domains and gene structure of the StDIR protein.

### 2.4 Phylogenetic analysis

To characterize the phylogenetic relationships of StDIR proteins, the amino acid sequences of DIR proteins from many plant species were downloaded from the TAIR database and Ensembl Plants database, including Arabidopsis and potato (*S. tuberosum*). In addition, a phylogenetic tree was also constructed in MEGA 7.0 with the neighbor-joining method. The Poisson model was selected and the boot value was set to 1,000.

### 2.5 Analysis of cis-acting elements in promoter regions of StDIR genes

The PlantCARE database (http://bioinformatics.psb.ugent.be/webtools/plantcare/html/) was used to predict the cis-acting elements in the promoter regions of *StDIR* genes. The promoter regions were analyzed using sequences 1.5 kb upstream of the initiation codon ATG. The cis-acting elements were classified into different groups based on their potential functions. Use the Python program to count the predicted results of cis-acting elements and use R v4.2 for visualization.

### 2.6 Duplication and Ka/Ks analysis of StDIR gene family

There are two values synonymous substitution rate (Ks) and nonsynonymous substitution rate (Ka) to describe the evolutionary relationship between genes (Hurst, 2002). The MCScanX-transposed software was used to identify the relationship between the *StDIR* gene family by using the CDS (coding sequence) sequences of 31 *StDIR* genes (Koch et al., 2000). The simple Ka/Ks calculator module of TBtools was used to calculate the value of Ka and Ks. At the same time, filter the results by the length of aligned genes was greater than 70% of the longer gene, and the similarity between the two genes was greater than 70%; the distance between the two genes was less than 100 kb (Vatansever et al., 2016). The diversity time was calculated by T = Ks/2r, and the r of dicotyledonous plants was taken to be 1.5 × 10^−8^ synonymous substitutions per site per year (Koch et al., 2000). The advanced circos module of TBtools was used to visualize collinear *StDIR* gene family members in the potato genome.

### 2.7 Synteny analysis of DIR genes in potato, arabidopsis, rice and tomato

To explore the synteny relationship of *DIR* genes between potato and Arabidopsis, rice, tomato, the genome sequence and genomic annotation file of Arabidopsis, rice and tomato were downloaded from the Ensembl plants database, and then the One Step MCScanX module of TBtools was used to comparative genomic analysis. The synteny relationship between the potato and the other three species was visualized by TBtools.

### 2.8 Gene expression analysis

In order to explore the response of the *StDIR* gene to high-temperature stress, the transcriptome data was used to analyze the expression profile of *StDIR* genes. The transcriptome data of accession: PRJNA577000 was downloaded from the NCBI (National Center for Biotechnology Information) database (https://www.ncbi.nlm.nih.gov/sra/). HISAT software was used to mapping the raw data to the potato genome (http://daehwankimlab.github.io/hisat2/), and the Rsubread package in R (version “4.3”) was used for the quantification of the gene (Mo et al., 2021). The expression of *StDIR* genes under high-temperature stress was analyzed by TBtools.

### 2.9 qRT-PCR analysis

Ten genes were selected to verify RNAseq. At the same time, after different stress treatments, we also carried out q-PCR of these ten genes to analyze the response of *StDIR* family members to stress. Primer 5 software was used for primer design (Table 1). Primers were ordered from Sangon Bioscience Co., Ltd. and the dye used for qRT-PCR was purchased from Biorun (BioRun Biosciences Co., Ltd., Wuhan, China). The system of qRT-PCR is referred to Mo’s study (Mo et al., 2021).

**TABLE 1:** The sequences of primers for qRT-PCR analysis.

Primer name	Primer sequences
StDIR1-RT-F	5′-TGG​AAG​GGC​ACA​AGG​GTT​TT-3′
StDIR1-RT-R	5′-ATC​GGA​AAA​GCC​CAC​TAC​CG-3′
StDIR2-RT-F	5′-CTG​CTT​CAC​TTA​ATG​ATG​TTG​GT-3′
StDIR2-RT-R	5′-ACA​GTA​GCA​TCT​CCA​GTT​TTG​A-3′
StDIR4-RT-F	5′-GGG​TCT​GCT​GGT​CAA​ACT​GA-3′
StDIR4-RT-R	5′-TGA​GCA​AGA​GCA​TAC​CCA​CG-3′
StDIR8-RT-F	5′-ATC​AGT​TGT​GGG​AGG​CAC​AC-3′
StDIR8-RT-R	5′-TGG​GAT​GAC​TTG​GAA​TGG​CA-3′
StDIR11-RT-F	5′-ATG​GCA​CCA​GCC​ACA​CTT​TA-3′
StDIR11-RT-R	5′-TGT​TGG​TCC​ACG​TGA​GGA​AG-3′
StDIR13-RT-F	5′-GCT​TCA​CAG​AGC​GAA​TCT​GC-3′
StDIR13-RT-R	5′-AGC​ATC​TCC​AGT​GTT​GGC​AT-3′
StDIR15-RT-F	5′-TAG​TTG​GAA​GAG​CGC​AAG​GG-3′
StDIR15-RT-R	5′-TCT​GAA​AAG​CCC​ACT​TCC​CC-3′
StDIR17-RT-F	5′-TGA​CTG​ATT​CCT​TTG​AGG​GTG​A-3′
StDIR17-RT-R	5′-CCT​ATT​ACC​CCA​ATC​CGG​CG-3′
StDIR22-RT-F	5′-TGC​ACA​AGG​TCC​TAA​AGC​TG-3′
StDIR22-RT-R	5′-TTC​TGG​TCC​CGT​TGT​CAA​TC-3′
StDIR25-RT-F	5′-GGT​TTG​TTG​CAA​TGG​CGG​AC-3′
StDIR25-RT-R	5′-TGG​GTT​CCG​ACC​AAG​TAC​AC-3′

## 3 Results

### 3.1 StDIR gene identification and chromosomal distribution analysis

To search for potato DIR proteins, the amino acid sequences of 25 Arabidopsis DIR proteins were used as blast queries against the total proteins sequence of potato. HMMER was used to analyze whether the protein contain the conserved DIR domain with the HMM file of PF03018. A total of 31 *StDIR* genes were identified in the potato genome (Table 2). The genome length of *StDIR* ranges from 443 (*StDIR7*) to 8,466 bp (*StDIR9*), and the genome length of 26 *StDIR* genes is less than 2,000 bp. The number of amino acids varies from 147 (StDIR7) to 708 (StDIR9), with 22 of the 31 StDIR proteins having less than 200 amino acids, suggesting that most StDIRs are small proteins. The molecular weight of the StDIR protein ranged from 16.31 kDa (StDIR7) to 79.60 KDa (StDIR9), and the theoretical pI ranged from 4.07 (StDIR11) to 10.28 (StDIR1). In addition, 18 StDIR proteins were predicted to be alkaline proteins (pI > 7.0) and 13 were predicted to be acidic proteins (pI < 7.0).

**TABLE 2:** The basic information of *StDIR* genes.

Gene name	Gene ID	Chr	Start	End	Aalen	MolWt	pI
*StDIR1*	PGSC0003DMT400082050	1	1576195	1577007	192	21259.8	10.28
*StDIR2*	PGSC0003DMT400013860	1	23816874	23817628	191	21315.66	9.83
*StDIR3*	PGSC0003DMT400092301	1	45553764	45554288	174	19398.43	7.09
*StDIR4*	PGSC0003DMT400004111	1	87245524	87246294	183	19380.17	7.5
*StDIR5*	PGSC0003DMT400004108	1	87247582	87248148	188	20761.87	9.54
*StDIR6*	PGSC0003DMT400038642	2	15631610	15632335	190	21103.36	9.25
*StDIR7*	PGSC0003DMT400038660	2	15661450	15661893	147	16310.66	8.82
*StDIR8*	PGSC0003DMT400003860	2	38554309	38555180	190	20825.83	8.91
*StDIR9*	PGSC0003DMT400075900	4	2743025	2751491	708	79607.82	5.63
*StDIR10*	PGSC0003DMT400077599	4	6664537	6665910	395	40792.31	4.3
*StDIR11*	PGSC0003DMT400060192	5	50524446	50526349	400	41211.73	4.07
*StDIR12*	PGSC0003DMT400060190	5	50527054	50534174	596	66343.27	8.55
*StDIR13*	PGSC0003DMT400086076	6	40684394	40684870	158	17339.67	7.07
*StDIR14*	PGSC0003DMT400078147	6	56302485	56303908	330	33175.07	4.43
*StDIR15*	PGSC0003DMT400035280	7	40214604	40215463	154	16827.42	9.64
*StDIR16*	PGSC0003DMT400074482	7	46761823	46762634	177	19599.45	6.89
*StDIR17*	PGSC0003DMT400031719	8	55448588	55451398	213	24204.43	6.23
*StDIR18*	PGSC0003DMT400030271	9	57735078	57737766	246	25708.18	4.83
*StDIR19*	PGSC0003DMT400058779	10	4515468	4516614	190	21328.81	10.23
*StDIR20*	PGSC0003DMT400056476	10	46575795	46576489	184	20239.66	9.56
*StDIR21*	PGSC0003DMT400056477	10	46576867	46577680	172	18875.67	4.87
*StDIR22*	PGSC0003DMT400056481	10	46580874	46581663	194	20933.17	9.59
*StDIR23*	PGSC0003DMT400095665	10	46589341	46589862	173	19227.94	5.54
*StDIR24*	PGSC0003DMT400056482	10	46593943	46594714	194	20913.23	9.65
*StDIR25*	PGSC0003DMT400056483	10	46623811	46624472	178	19489.43	9.9
*StDIR26*	PGSC0003DMT400095095	10	46642431	46642955	174	19347.05	6.23
*StDIR27*	PGSC0003DMT400056484	10	46659222	46659794	190	20945.13	8.92
*StDIR28*	PGSC0003DMT400056489	10	46673839	46677348	150	16314.58	5.82
*StDIR29*	PGSC0003DMT400059698	10	46701611	46702266	168	18411.4	10.12
*StDIR30*	PGSC0003DMT400040204	11	45222690	45223700	336	34327.28	4.53
*StDIR31*	PGSC0003DMT400022230	12	58771170	58772579	247	25745.46	5.13

Chr, chromosome number; Aalen, amino acid length; MolWt, molecular weight; pI, isoelectric point.

**FIGURE 1:**
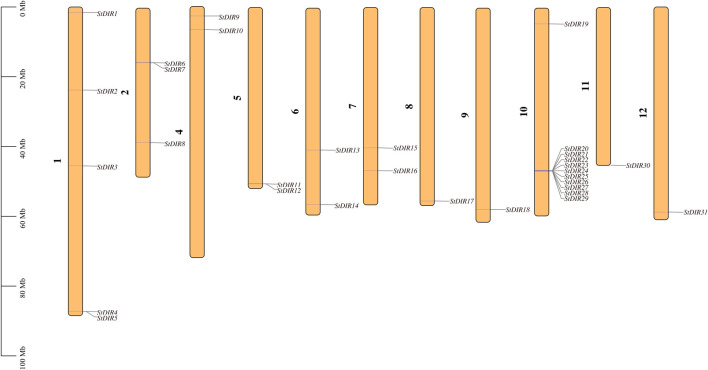
Chromosomal distributions of *StDIR* genes. Chromosome number is indicated.

A total of 31 *StDIR* genes were identified on 12 potato chromosomes and their distribution on chromosomes was uneven. Except for chromosome 3, all 11 chromosomes contained the *StDIR* genes (Figure 1). Among these chromosomes, chromosome 10 contains the most *StDIR* genes, with 11 members, and the positions of these genes on chromosome 10 are very close, except for *StDIR19*. Chromosome 1 is followed by four members. The distribution of these 31 *StDIR* genes on chromosomes is not obvious, some are distributed in the middle of the chromosome (near the centromere), and some are distributed at both ends of the chromosome.

### 3.2 Phylogenetic relationships among StDIR genes

**FIGURE 2:**
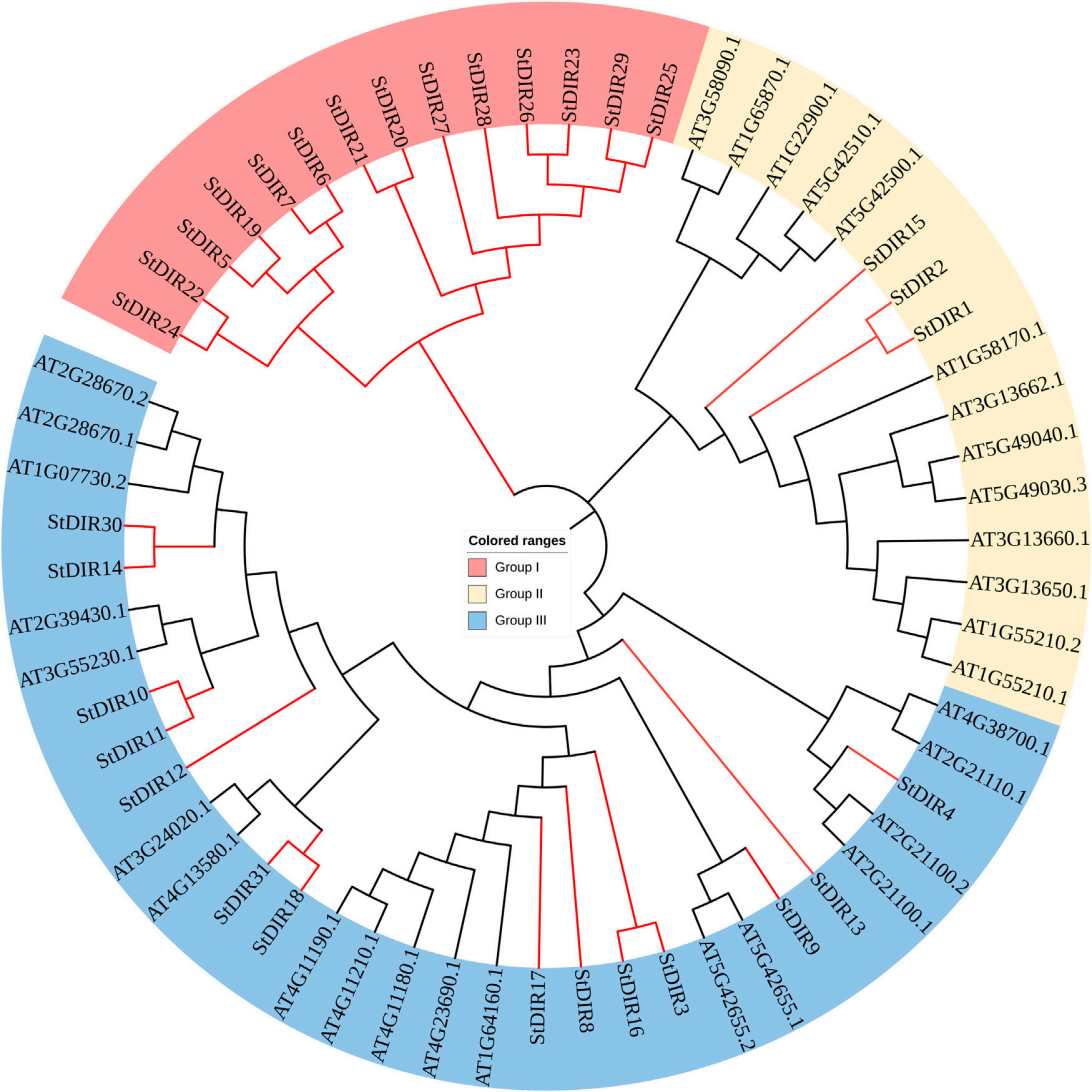
Phylogenetic tree of DIR proteins in Arabidopsis and potato.

To group *StDIR* genes into different subfamilies and predict the functions of *StDIRs* from well-studied *DIR* homologous genes in other species, the amino acid sequences of DIR proteins from Arabidopsis were used to construct a phylogenetic tree using MEGA 7.0. Our analyses showed that *DIR* gene families were divided into three major subclades, of which group III had the most DIR proteins, while Group I all belonged to potato, with a total of 14 proteins. 3 potato proteins and 13 Arabidopsis proteins belong to Group II, and 14 potato proteins and 18 Arabidopsis proteins belong to Group III (Figure 2). It is worth noting that the phylogenetic tree found that Group III contains a large number of StDIR and AtDIR proteins, indicating that they are highly conserved during evolution and may have similar functions.

### 3.3 Conserved motifs, conserved domain and gene structure analyses of StDIR genes

**FIGURE 3:**
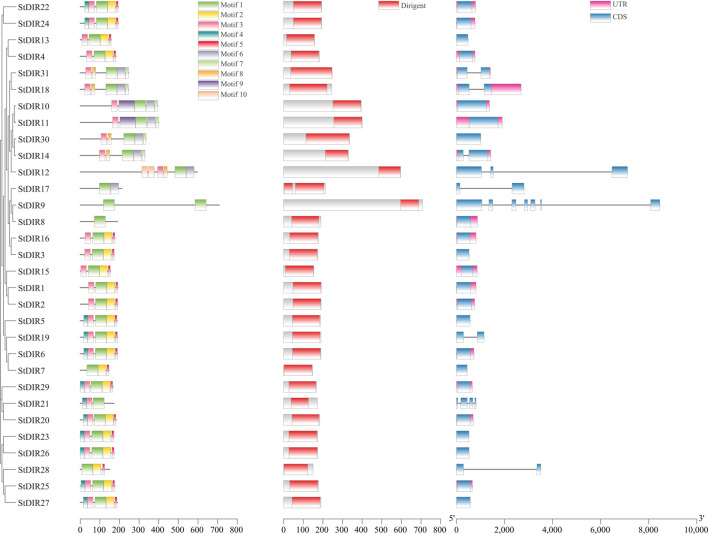
Conserved motifs, conserved domain and gene structure of potato DIR proteins.

To study the exon-intron organization of the *StDIR* genes, we used the gene Structure Display Server program to analyze the *StDIR* genome and coding sequence. Of the 31 *StDIR* genes, 22 had classical *DIR* gene structure, which has one exon and no intron. The exception is *StDIR31*, *StDIR18*, *StDIR14*, *StDIR12*, *StDIR17*, *StDIR9*, *StDIR19*, *StDIR21*, and *StDIR28*, there are one or more introns (Figure 3). In addition, most *StDIR* genes contain UTRs, 17 in total, and *StDIR13*, *StDIR30*, *StDIR12*, *StDIR17*, *StDIR9*, *StDIR3*, *StDIR5*, *StDIR19*, *StDIR7*, *StDIR21*, *StDIR23*, *StDIR26*, *StDIR28* and *StDIR27* has no UTRs (Figure 3). The conserved DIR domain accounts for the majority of proteins in most StDIR except StDIR9, StDIR10, StDIR11 and StDIR12. Then, we used the MEME tool to analyze the conserved motif of the StDIR protein. A total of 10 distinct motifs were detected in all 31 StDIR proteins. All 31 StDIR proteins contain the conserved DIR domains: Dirigent domain. The difference in motifs reflects the diversity of StDIR proteins. For example, motif 9 only exists in StDIR10 and StDIR11, motif 10 only exists in StDIR12, and motif 8 only exists in StDIR12, StDIR14, StDIR18, StDIR30 and StDIR31, indicating that the StDIR protein has various functions.

### 3.4 Duplication analysis of potato DIR genes

**FIGURE 4:**
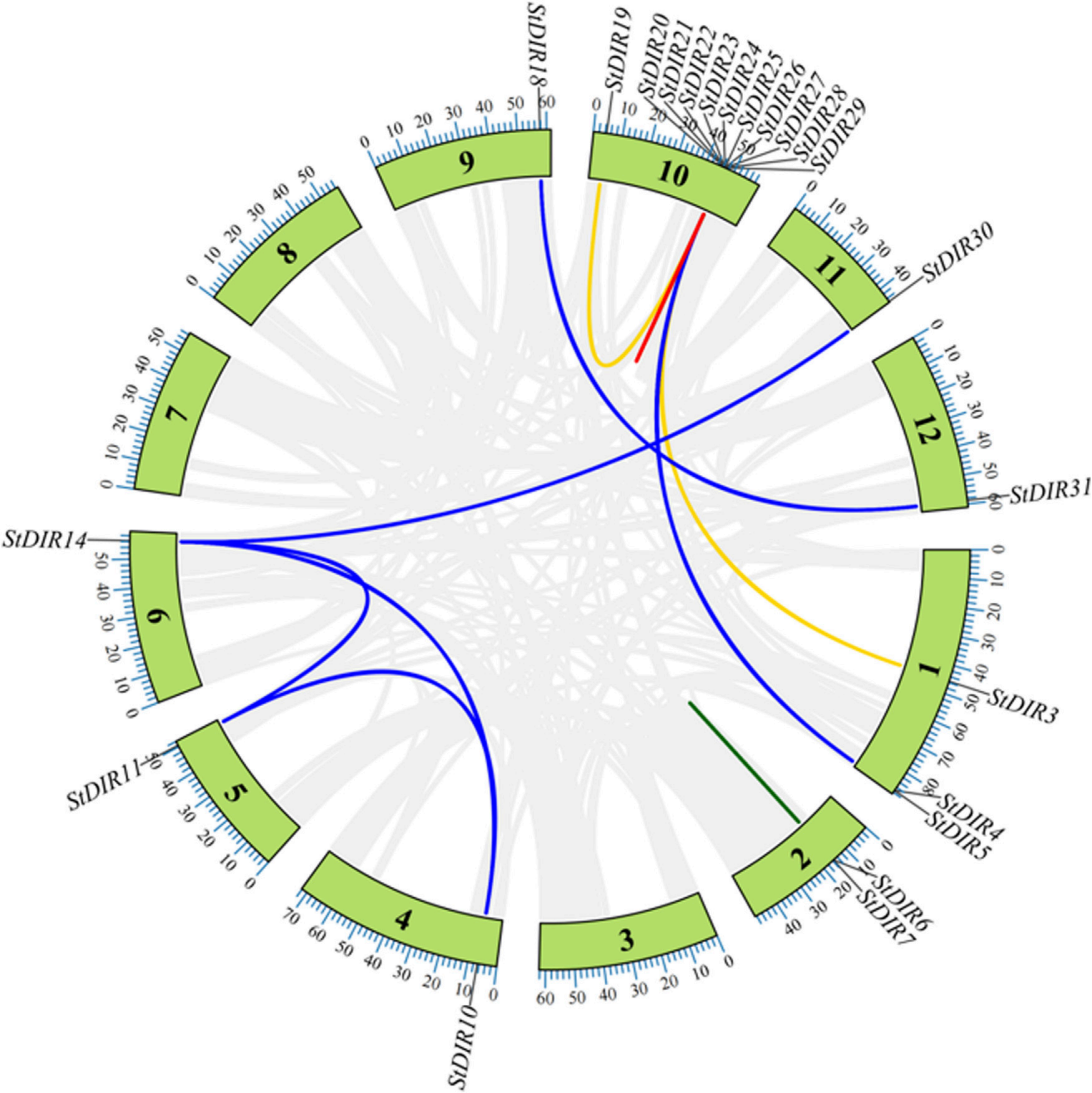
The duplication genes in the *StDIR* gene family. Proximal duplication gene pairs are inked by the red line, proximal duplication gene pairs are linked by the blue line, tandem duplication gene pairs are linked by the green line and transposed duplication gene pairs are linked by the yellow link. And there are too many pairs in Chr 10, so there only one line can be shown in the figure.

As shown in Table 3, there are a total of 22 *StDIR* gene family members with four duplicate relationships: proximal duplication (3 gene pairs, 5 *StDIR* genes), segmental duplication (7 gene pairs, 10 *StDIR* genes), tandem duplication (4 gene pairs, 8 *StDIR* genes) and transposed duplication (2 gene pairs, 3 *StDIR* genes) have been identified in *StDIR* gene family. The value of Ka/Ks were between 0.0987 (StDIR10 and StDIR11) and 0.5101 (*StDIR28* and *StDIR29*), all the *StDIR* duplicate genes with a Ka/Ks value less than 1, means all the genes in Table 3 were purifying selection. And the divergence time of *StDIR* duplicate genes range from 19.6013 Mya to 1320.6681 Mya. The genomic position of all the *StDIR* genes with duplication relationship were shown in Figure 4.

**TABLE 3:** The duplication gene pairs in StDIR gene family.

Duplicate 1	Duplicate 2	Mode	E-value	Ka	Ks	Ka/Ks	Divergence time (Mya*)
*StDIR22*	*StDIR24*	proximal	7.12E-135	0.0115	0.0653	0.1760	19.6013
*StDIR23*	*StDIR25*	proximal	3.02E-95	0.1495	0.4339	0.3446	130.1701
*StDIR25*	*StDIR26*	proximal	9.63E-97	0.1389	0.4262	0.3258	127.8687
*StDIR4*	*StDIR24*	segmental	2.16E-51	0.5416	1.6498	0.3283	494.9253
*StDIR5*	*StDIR25*	segmental	2.3E-68	0.2993	2.4080	0.1243	722.4091
*StDIR30*	*StDIR14*	segmental	1.98E-145	0.1594	0.9538	0.1671	286.1374
*StDIR31*	*StDIR18*	segmental	4.34E-139	0.1017	0.5849	0.1738	175.4680
*StDIR10*	*StDIR11*	segmental	2.95E-172	0.1344	1.3610	0.0987	408.2912
*StDIR10*	*StDIR14*	segmental	9.82E-69	0.4474	1.9013	0.2353	570.3921
*StDIR11*	*StDIR14*	segmental	2.5E-67	0.4712	4.4022	0.1070	1320.6681
*StDIR20*	*StDIR21*	tandem	1.04E-61	0.1311	0.2649	0.4948	79.4629
*StDIR26*	*StDIR27*	tandem	2.86E-96	0.1366	0.4062	0.3364	121.8642
*StDIR28*	*StDIR29*	tandem	9.93E-65	0.1267	0.2484	0.5101	74.5334
*StDIR6*	*StDIR7*	tandem	1.76E-96	0.0465	0.2451	0.1896	73.5380
*StDIR19*	*StDIR25*	transposed	8.48E-81	0.2605	1.3264	0.1964	397.9113
*StDIR3*	*StDIR25*	transposed	2.86E-32	0.6349	2.0847	0.3046	625.4146

Mya: million years ago.

### 3.5 Promoter cis-element analysis of StDIR genes

**FIGURE 5:**
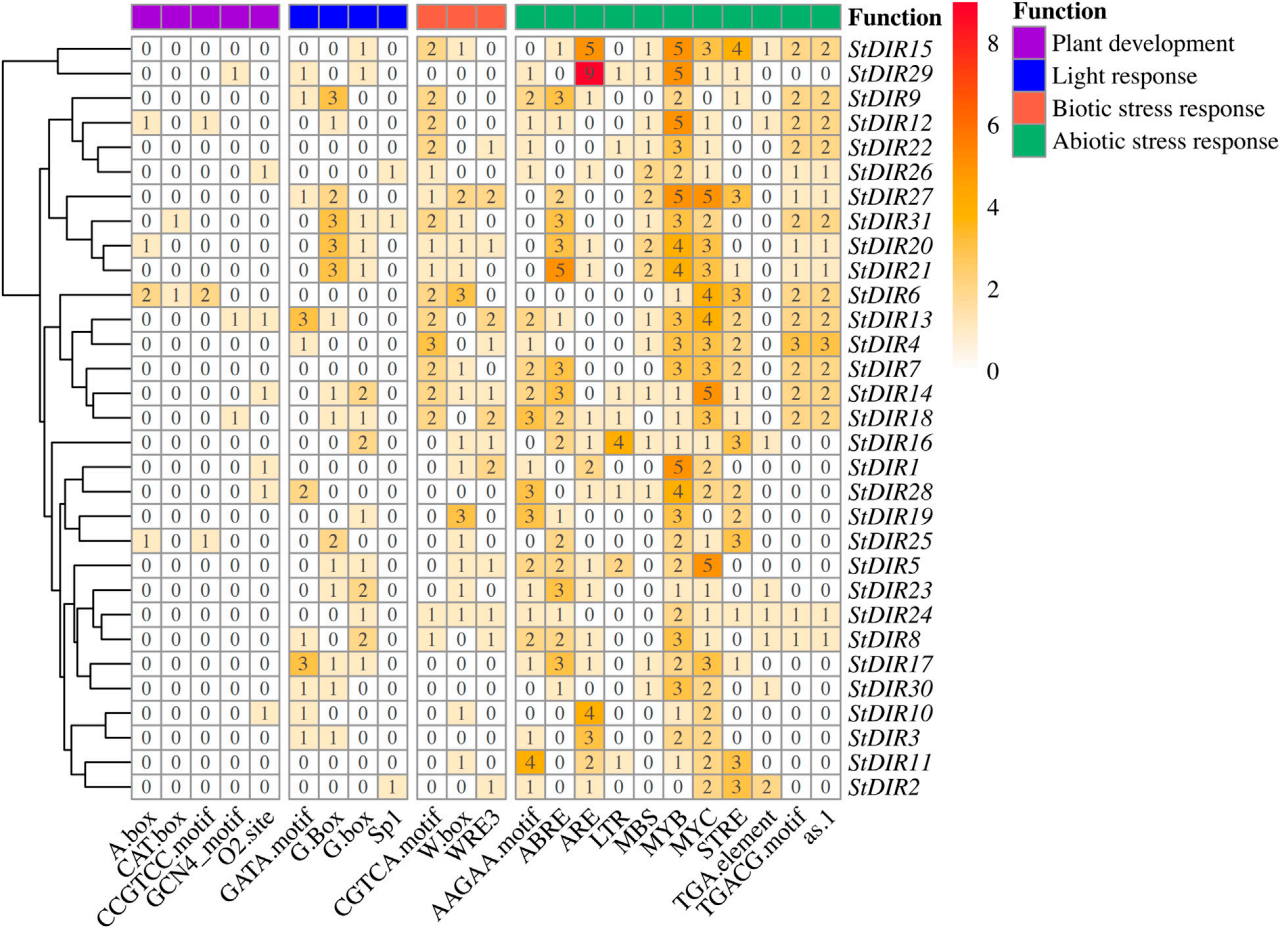
Cis-acting element analysis in *StDIR* promoter regions.

To reveal the diversity of cis-elements in the promoter region of *StDIR* genes, we selected the 1500 bp upstream *StDIR* gene sequence and submitted it to the PlantCARE database for promoter cis-element analysis. R package pheatmap was used to create heatmaps to visualize the four classes of cis-components (Figure 5). Among them, 5 components were related to plant development, 4 components were related to light response, and 3 components were related to biotic stress. The largest number of elements is related to abiotic stresses, with a total of 11 elements. This indicates that the *StDIR* gene family is mainly involved in the regulation of abiotic stress in plants. For example, LTR is a group of low-temperature response elements, and MBS is a drought response element. Among the abiotic stress-related elements, the number of members of the *StDIR* family containing MYB is the largest. Among the 31 members, only *StDIR2* does not have a MYB element. The MYB element has a wide range of functions and responds to abiotic stresses such as high temperature, drought, low temperature and high salt. In addition, the number of abiotic stress elements identified in the promoter region of *StDIR* was second only to biotic stress, including CGTCA-motif, W box and WRE2. A total of 26 family members contained 68 biotic stress elements, indicating that *StDIR* family members also play an important role in plant biotic stress. There are also a large number of light response elements and plant development elements in the *StDIR* family, but the number is far less than biotic stress and abiotic stress elements. This elucidates the pivotal role of StDIR in orchestrating crucial responses to both biotic and abiotic stresses in plants.

### 3.6 Synteny analysis of DIR genes between potato and arabidopsis, rice, tomato

**FIGURE 6:**
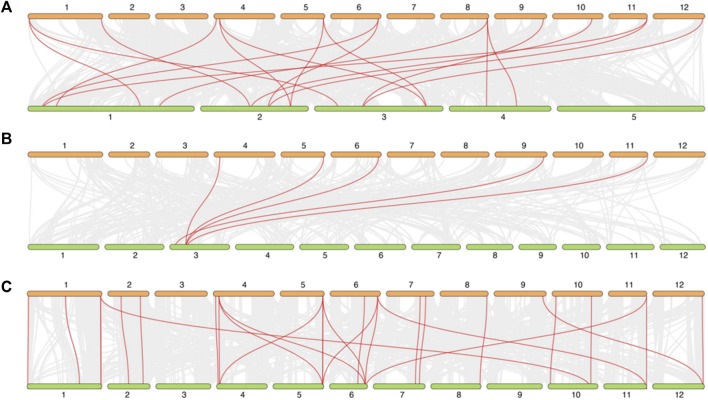
The synteny relationship between potato and Arabidopsis, rice, tomato. **(A)** potato and Arabidopsis, **(B)** potato and rice, **(C)** potato and tomato. All the synteny relationship *DIR* genes between different species were linked by the red line, the collinear blocks within the potato and other plant genomes were linked by the gray lines in the background.

To explore the synteny relationship between *DIR* genes in different species, three species were selected, containing the model and dicotyledons plant: Arabidopsis, monocotyledon and crop plant: rice and the Solanaceae plant: tomato. As shown in Figure 6, there are 18 *DIR* gene pairs (involving 11 *StDIR* genes) between potato and Arabidopsis, 5 *DIR* gene pairs (involving 5 *StDIR* genes) between potato and rice and 28 *DIR* gene pairs (involving 20 *StDIR* genes) between potato and tomato.

### 3.7 Expression pattern analysis of potato StDIR gene family members under stress

**FIGURE 7:**
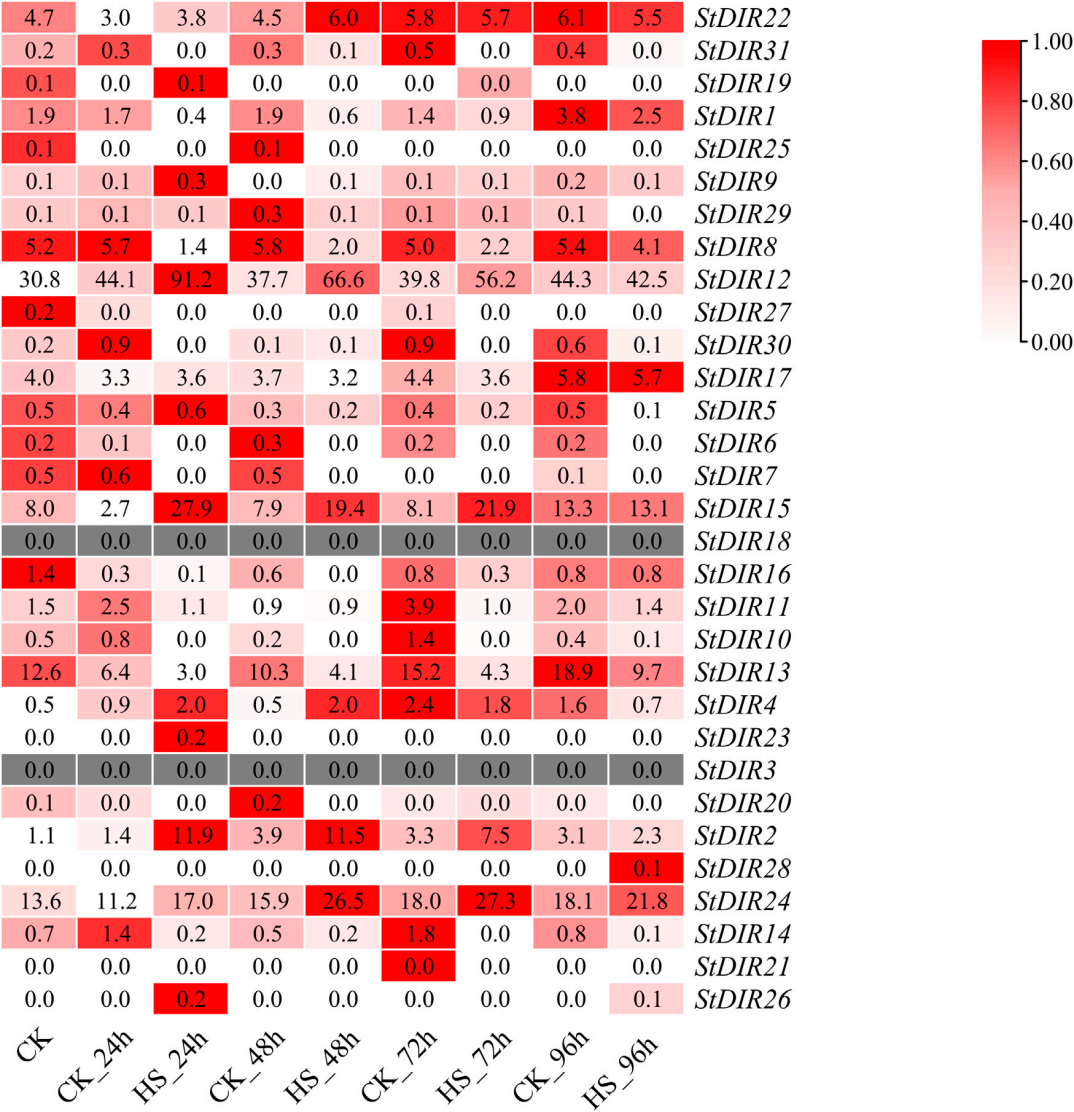
Relative expression levels of *StDIR* genes in transcriptome.

In the transcriptome data, *StDIR12* had the highest expression level after 24 h of heat stress. Subsequent to heat stress, the StDIR family members primarily exhibited two distinct trends: an initial decrease followed by an increase, and a gradual increase (Figure 7). Among them, *StDIR22*, *StDIR31*, *StDIR1*, *StDIR30*, *StDIR17*, *StDIR11*, *StDIR13* and *StDIR14* showed a trend of decreasing first and then increasing, while StDIR12, StDIR15, StDIR4, StDIR2 and StDIR24 were significantly increased after heat stress. Concurrently, StDIR3 and *StDIR18* were not discernible within the transcriptomic dataset. These results indicated that most members of the *StDIR* family responded to heat stress to some extent, indicating that the *StDIR* gene family may play an important role in plant response to heat stress.

**FIGURE 8:**
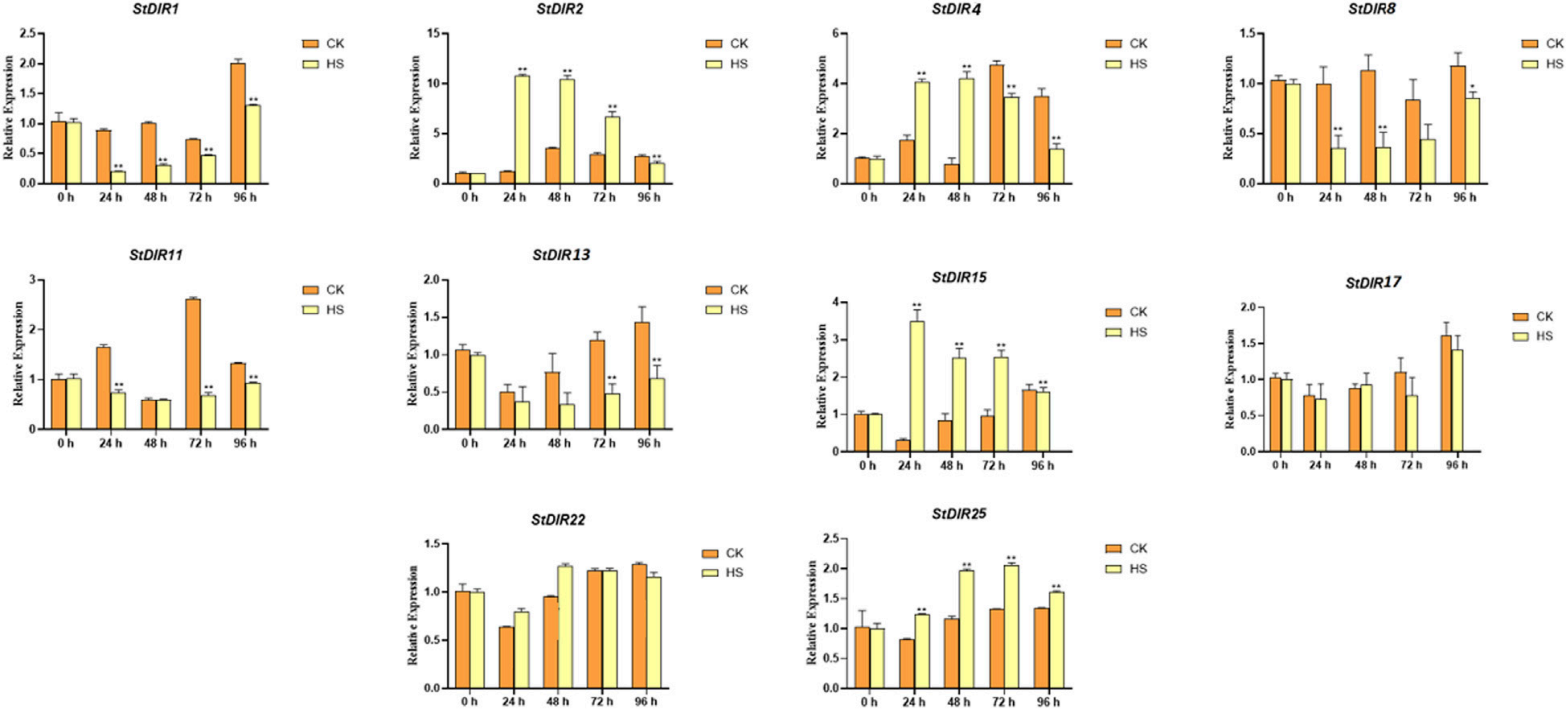
The expression of the ten genes selected in the transcriptomic data. HS means high temperature stress.

To verify the accuracy of RNA-Seq data and study the response of the *StDIR* gene family to abiotic stress in potato, ten *StDIR* family members, *StDIR1*, *StDIR2, StDIR4, StDIR8, StDIR11, StDIR13, StDIR15, StDIR17, StDIR22* and *StDIR24* were selected in this study. As shown in Figure 8, the chosen genes exhibited an initial decrement followed by an increment under heat stress, aligning seamlessly with the transcriptome data findings.

**FIGURE 9:**
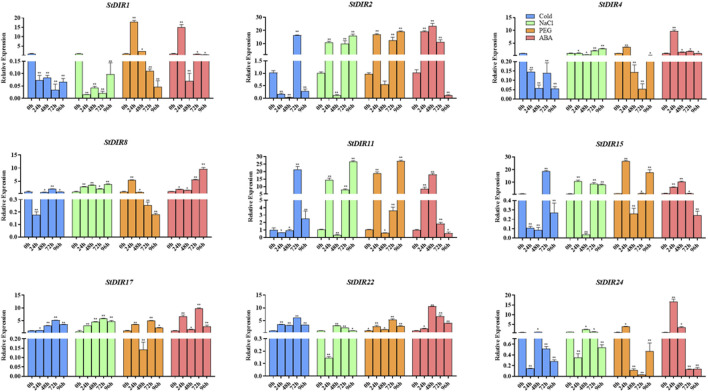
Expression levels of nine selected *StDIR* genes in response to cold stress, salt stress, drought stress and ABA treatment.

Then, we treated potato plants with cold stress, salt (NaCl) stress, drought (PEG) stress, and ABA treatment and selected nine genes from different subclades of the phylogenetic tree to analyze their responses (Figure 9). Under cold stress, *StDIR22*, *StDIR25* and *StDIR1* were significantly downregulated, while *StDIR2, StDIR11* and *StDIR15* was significantly upregulated. Under salt stress, *StDIR2, StDIR11, StDIR15, StDIR22* and *StDIR25* were significantly upregulated but had no obvious trend, while *StDIR1* was significantly downregulated. After ABA and PEG treatment, the overall trend of the selected genes increased first and then decreased. The foregoing outcomes signify the responsiveness of StDIR family constituents to a majority of stresses or treatments, further substantiating the crucial role the gene family may play in plants' reaction to diverse abiotic stresses.

## 4 Discussion

Abiotic stress is an environmental adversity that affects the normal growth and development of plants, including drought, high salt and extreme temperature, and is the main factor affecting crop yield. Abiotic stress inhibits plant photosynthesis, affects chloroplast stability and induces chloroplast degradation, thus causing premature senescence of plants and ultimately affecting crop yield (Khatun et al., 2017; Ogden et al., 2020; Liu et al., 2022). The damage caused by inhospitable environmental conditions has severely limited the yield of potato, so the mining of excellent resistance genes in plants has become one of the main methods of researchers today.

Over the past few decades, several studies have characterized the *DIR* gene, and this work has greatly increased our understanding of plant responses to stress in many species. Many studies of *DIR* gene expression have associated its involvement with biological and abiotic stresses. However, until now, the *DIR* gene family has not been reported in potato, which has hindered the research on the function of the potato *DIR* gene to some extent. Based on this, a systematic bioinformatics analysis of the potato *DIR* gene family was carried out in this study. In this study, 31 *DIR* genes were identified from potato, more than those from pepper (*Capsicum annuum*) (Khan et al., 2018) and Brassica (*Brassica rapa*) (Arasan et al., 2013), indicating that potato contains more family members, and their expression patterns under heat, cold, salt and drought stress were analyzed. The 31 *StDIR* genes in the potato genome have an average molecular weight of 25.8 KDa and an average pI of 7.51 (Table 2). Meanwhile, we also carried out chromosome localization of the potato *StDIR* gene family, constructed the evolutionary tree of potato and Arabidopsis, and explored the phylogenetic relationship of the potato *DIR* gene family with Arabidopsis as a reference. In addition, four classes of cis-acting elements were identified in the analysis of the *StDIR* gene family, which were related to plant development, light response, abiotic stress and biological stress. Among these elements, most elements are associated with abiotic stress. Wu et al. found that in Ulva prolifera (Ulvophyceae, Chlorophyta), both HSE and LTR are indispensable under cold stress, as the deletion results in a complete loss of promoter activity. In algae domestication and artificial breeding, HSE and LTR elements may be potential molecular targets for temperature domestication (Wu et al., 2019; Puchtel, 2022). It was reported that 65% of MYB genes expressed in seedlings were differentially regulated under drought stress in rice (Katiyar et al., 2012; Peng et al., 2022; Qu et al., 2022). An even higher percentage is observed in Arabidopsis: transcriptomic data collected in the GENEVESTIGATOR database (Zimmermann et al., 2004a; Zimmermann et al., 2004b) show that 51% of *AtMYB* genes are upregulated by drought and 41% are downregulated (Katiyar et al., 2012). In the analysis of cis-acting elements, elements related to biological stress such as WRE3, CGTCA-motif and W Box also accounted for a large part. The existence of such a large number of biological and abiotic stress response elements further proves that the *StDIR* gene family plays a key role in potato coping with biological and abiotic stress.

Gene duplication plays an important role in gene diversity, *StDIR25* can be found in proximal duplication, segmental duplication and transposed duplication with a total of 5 times, *StDIR25* may be a key gene in the gene duplicate events (Figure 4).

The synteny relationship genes analysis between different species can show that the diverge of genes followed by the diverge of species. In Figure 6, monocotyledon plant rice had a long divergence time with potato, so there are only 5 pair homologous, 18 pair homologous in dicotyledons Arabidopsis, and there 28 pair homologous between Solanaceae plant potato and tomato, The results indicated that the *DIR* genes are more homologous and conserved in dicotyledons than monocotyledons, and Solanaceae than dicotyledons. Besides, there are not only the number of homologous is the most in potato and tomato, but also the homologous have the parallelism between *DIR* genes on chromosomes, such as chromosome 2.

Subsequently, we analyzed the expression levels of the potato *StDIR* gene family after heat stress by transcriptomic data and selected four genes for q-PCR. The results showed that the q-PCR trend of the selected genes was the same as that of the transcriptome. This also verified the authenticity and reliability of transcriptome data. In the whole transcriptome, most members of the *StDIR* gene family showed a response to heat stress. Most members of the *StDIR* gene family showed an increasing trend after plants were treated with heat stress. We then treated potatoes with cold, salt, ABA and PEG, and selected some genes for qRT-PCR detection to further explore the response of the *StDIR* gene family to different stresses. Through the above experiments, we once again verified that the *StDIR* gene family has a certain degree of response to many abiotic stresses, indicating that the members of the *StDIR* gene family play a key role in the process of potato abiotic stress. It has been shown that the participation of *AtDP1*/*AtDIR12* and *AtLAC5* in the biosynthesis of new lignans through SC/FC has a strong protective effect on Arabidopsis seeds (Yonekura-Sakakibara et al., 2021). Furthermore, both the stress tolerance from *ScDIR*-expressed *E. coli* and its increased expression in sugarcane suggest that the *ScDIR* gene is involved in the response to abiotic stresses of drought, salt and oxidant (Guo et al., 2012; Li et al., 2022). Ralph et al. revealed a role for spruce *DIR* genes in constitutive and induced phenolic defense mechanisms against stem-boring insects (Ralph et al., 2006).

DIR not only plays an important role in a variety of biological and abiotic stresses, but also as a key gene in many other aspects due to its special gene function. The stone cell is a special cell of pear, which is a kind of dead cell formed by the lignification of the thin-walled cell wall in the pulp (Lu et al., 2015; Zeng et al., 2022). It can seriously affect the pear quality and economic benefits (Thompson et al., 2003; Sugino et al., 2018; Zhang et al., 2022; Wang et al., 2022). Therefore, regulating the content of stone cells is the key to improving the pear quality. Cheng et al. found that *PbDIR* may also be involved in stone cell development and lignin metabolism of pear (Jiang et al., 2020). Keiko et, al. found that *AtDP1*/*AtDIR12* and *AtLAC5* are involved in neolignan biosynthesis via SC/FC. A tetrazolium penetration assay showed that seed coat permeability increased in atdp1 mutants, suggesting a protective role of neolignans in Arabidopsis seeds (Yonekura-Sakakibara et al., 2021). Studies have also shown that the *DIR1* gene promoter could be activated by MeJA, NaCl, and D-mannitol, and inhibited by ABA (Roh et al., 2010; Li et al., 2021). By analyzing the response of DIR-promoter to different hormones in different species, Li et, al. revealed the regulatory mechanism of the *DIR* gene to a certain extent, which played a certain reference role in the study of the specific mechanism of *DIR* gene family members. Lignin is closely related to *DIR*. Modern agricultural and industrial processes generate a significant amount of lignin-based waste, most of this involving use of fungal-mediated bio-catalysis (Lo et al., 2021; Maldhure et al., 2022; Pongchaiphol et al., 2022). Siarhei studied the *DIR* family of bacteria and suggested that bacteria may be better at degrading lignin than traditional fungi due to differences in *DIR* family members (Dabravolski, 2020). The *DIR* gene family has prominent functions in many aspects. Bioinformatics analysis of the *DIR* gene family and analysis of its gene structure can provide a strong reference for an in-depth exploration of its functions.

## 5 Conclusion

In summary, this study identified *StDIR* gene family members in potatoes for the first time. Through bioinformatics analysis and qRT-PCR analysis under abiotic stress, it revealed that *StDIR* gene family members in potatoes may play an important role in potato growth and development, stress and hormone response. This study provides ideas for studying the response of the *StDIR* gene in potato to stress and also provides a basis for further studies on the role of the *StDIR* gene family in potato breeding and resistance improvement.

## Data Availability

Publicly available datasets were analyzed in this study. This data can be found here: NCBI (National Center for Biotechnology Information) database (https://www.ncbi.nlm.nih.gov/sra/), PRJNA577000.
